# Interpretation of the adsorption of metals on quartz crystal based-macromolecule via advanced modeling of equilibrium isotherms

**DOI:** 10.1038/s41598-021-99465-9

**Published:** 2021-10-08

**Authors:** Fatma Aouaini, Mohamed Ben Yahia, Haifa I. Alrebdi, Miysoon A. Alothman

**Affiliations:** 1 Department of Physics, College of Science, Princess Nourah Bint Abdulrahman University, Riyadh, Saudi Arabia; 2 Laboratory of Quantum and Statistical Physics LR18ES18, Faculty of Sciences of Monastir, 5000 Monastir, Tunisia

**Keywords:** Biochemistry, Biophysics, Chemistry, Engineering, Physics

## Abstract

In this article, new insights about the metals-porphyrin complexes are proved by analyzing the zinc, nickel and chromium adsorption process over the well-known porphyrin macromolecule. The use of the quartz crystal microbalance (QCM) apparatus allows the control of the complexation systems’ experimental adsorption data operating at four temperatures. The experimental results and the physical models reveal that the zinc and nickel complexation processes are to be examined using the mono layer adsorption model. While, the double layer model describes the interaction between the chromium compound and the porphyrin. Actually, the three metals are shown to be adsorbed by a multi-docking process in the physicochemical description. The endothermic character of the investigated processes is shown through the appropriate data of the principal parameter adsorbent sites’ density. Hence, several porphyrin sites are exclusively stimulated at high temperature. The parameters of van del Waals, depicting the influences of the lateral interactions, explain the nickel isotherms down trend. The chemical bonds are shown to be carried out between the zinc and the porphyrin through the calculated adsorption energies. Considering the thermodynamic study, and referring to the configurational entropy and the free enthalpy, it is to be noted that the disorder peak of the three mechanisms is reached when the equilibrium concentration is equal to the energetic parameters’ values for each system. The nickel enthalpy revealed for high concentration that the adsorbates’ lateral interactions disapproved the nickel chloride adsorption. The free enthalpy trends, that observed two stability states of the chromium compound, confirmed the chromium double layer mechanism.

## Introduction

The macrocyclic components known as porphyrins have been playing important role in biological processes in the field of the living organisms metabolism such as the photosynthesis mechanism and the oxygen transport process since life-system is formed^1–3^. Thus, the hemoglobin and the chlorophyll structures showed the compounds of porphyrins with magnesium and iron. Therefore, the macrocyclic complexes are called pigments of life^4^. Moreover, the aggregation of porphyrins is studied intensively because the change in their microscopic characteristics led to their application in several research areas such as the artificial photosynthetic systems, the sensor devices and the nonlinear optical materials …^5^. Recently, a monumental importance has been given to the complexation of these chemical elements by various metals. This complexation allowed the birth of different metalloporphyrins compounds^6–8^. The sensing properties as well as the photodynamic activity are favored by the complexed metals^9–13^. For instance, metalloporphyrins are used as ionophores when it comes to ion selective sensors^9^. The metallo-porphyrins of aluminum and manganese contributed as well to the fields of optical anion sensors and potentiometric^14–18^. The cobalt-porphyrin photosensitizing properties are proved to be of much use in the photodynamic therapy as well^4^. The rising importance given to the chemical complexes resulted in an evaluation of porphyrin complexes along with indium and aluminium from different areas such as the stereochemistry, the polymerisation reactions, the molecular recognition field and the supramolecular building blocks^19–22^. Late research papers have proved that even the enzymatic reactions study involved the applicability of the complexes of porphyrin-based metals like the complexes of porphyrin with nickel and chromium^23,24^. Metalloporphyrins were also investigated in the field of anticancer activities where the zinc-porphyrin complexes have demonstrated potential against fungal growth^25^ and in other areas of bioelectronics and organic electronics based-devices^26^.

These important mechanisms of metalloporphyrins made researchers inspect the synthesis mechanism of these compounds by various experimental approaches^27,28^. Notably, late works have inspected the phenomenon of electrodes’ coating with polymers. The electrode based polymers have been framed via spin-coating on the electrode surface or by a preformed polymer application. They have been proven to be applicable in multiple frameworks such as biosensors, molecular electronics and electrocatalysis etc.…^29,30^. Importantly, gold electrode based porphyrins film has gained potential interest^11^. This way, our research team opted for the quartz-crystal-microbalance (QCM) technique to make use of the complexion mechanism of porphyrins by various metals. The spin coating process is selected for the deposition of porphyrins on the quartz crystal electrode. After that, the metal’s mass on the functionalized crystal for every concentration was controlled through the QCM technique, which leads to scheme the curves of the isotherms at different temperatures^31^.

**Figure 1:**
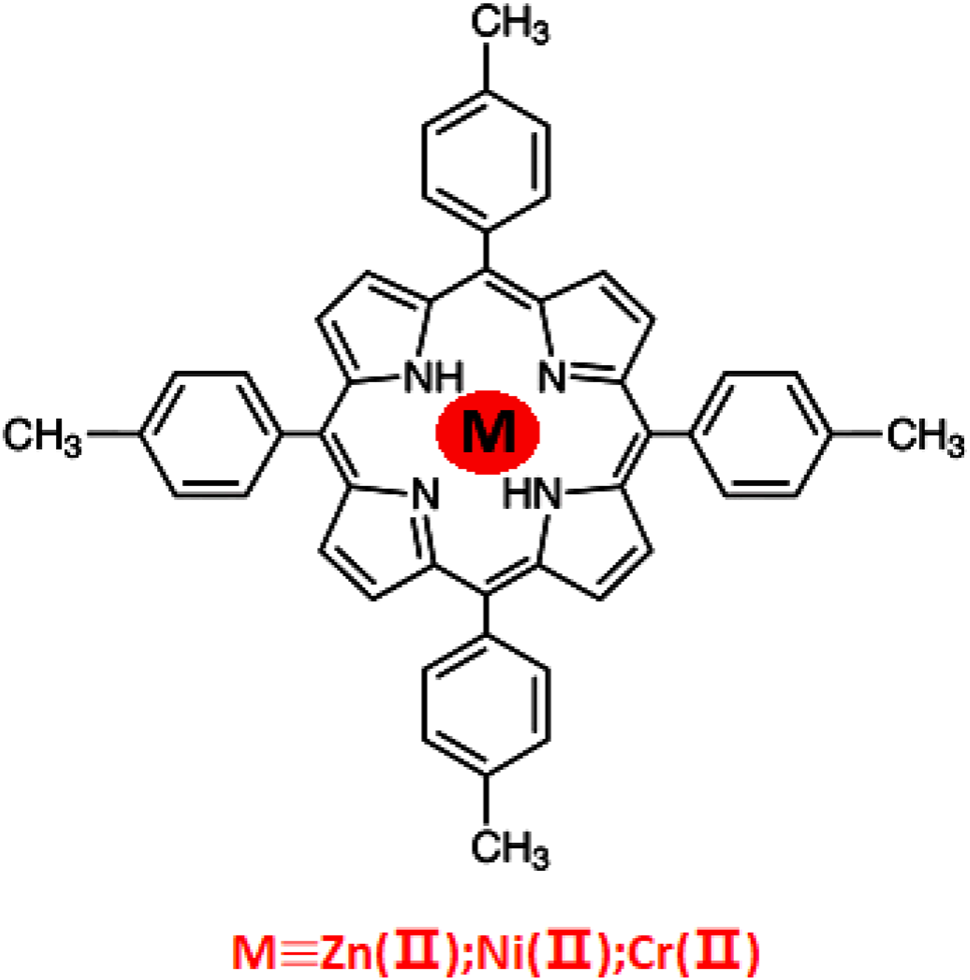
Chemical structure of the complexes of the 5,10,15,20-tetrakis(4-methylphenyl) porphyrin with the three metals (zin, nickel and chromium).

In this paper, the QCM method is devoted for the complexation mechanism study of 5,10,15,20-tetrakis(4-methylphenyl) porphyrin with three metals (zinc, nickel and chromium) (Fig. 1). The selection of these three complexes was due to two reasons: on the one hand, their fields of application were interesting ones. On the other hand, they were not well inquired beforehand. The adsorption’s analysis of some compounds of these metals on the porphyrinic surface is required in the study of the process of complexation by means of the QCM technique. In fact, the adsorption isotherms of zinc chloride, nickel chloride and chromium chloride on the quartz sensor coated with the macromolecule should be performed at different reaction temperatures.

The empirical equations as the well-known Langmuir equation or the Freundlich model could pave the way for an interpretation of adsorption isotherms^32,33^. Although the previously mentioned models are selected in order to examine numerous adsorption problems, they did not lead to satisfactory energetic or steric interpretations^32–35^. In recent years, the introduction of the ideas of the statistical physics treatment has improved the descriptive models progress, which led to advanced equation of models^36,37^. The advanced models contribution in both energetic and steric adsorption phenomenon- analysis, by means of the models’ variables, came up with an interesting description of an activated surface at the ionic scale. This fact requires the choice of the most suitable model that is the appropriate one for experimental adsorption data fitting.

This paper is devoted to discuss the following objectives:

- The performance of the tetrakis(4-methylphenyl) porphyrin which acts as sensor of the three metals is checked by the application of the QCM technique for the measurement of isotherms plots.
- The experimental results’ description is presented via the statistical physics theory.
- The appropriate descriptive models are selected through numerical simulation.
- At the ionic level, the energetic and the steric interpretations of the complexation process behavior are developed considering the adopted model parameters.
- The adsorption energies’ calculation is carried out to estimate the metal porphyrin bond’s nature.
- The macroscopic discussion concerning the adsorption of the three ions is done based on a thermodynamic study^38^.

## Experimental adsorption measurements’ method

### Piezoelectric sensor setup

The QCMs are improved techniques of mass-measurement at the nanogram-scale^39–42^, based on the quartz-inherent property of piezoelectricity. The quartz ‘AT-cut’, represents one of the most suitable cut giving satisfactory stability properties, the crystal quartz sensor is characterized by a frequency order of MHz. This work is carried out considering a frequency *f*_*0*_ of 5 MHz with a coinciding crystal’s thickness equal to 330 μm. The disc diameter which is about 2.54 cm is covered with a gold layer. The area of this latter is characterized by an upper electrode of 1.37 cm^2^ and a lower electrode of 0.4 cm^2^^40^. The quartz discs are cleansed using hydrogen peroxide as well as with sulfuric acid at room temperature. Later, deionized water and ethanol are used to rinse them. In attempt to get rid of any remaining water, the crystals are dried using high purity nitrogen.

Before film deposition, the porphyrin compound was dissolved in chloroform resulting in a solution of concentration equal to 2.9 10^–2^ mol L^−1^. Then, the gold-quartz electrode functions as a spin-coating 40 μL of porphyrin solution is performed onto the crystal surface at a speed of 3000 rpm during 30 s. A temperature of 100 °C is used to dry up the adsorption cell or the porphyrin coated on quartz crystal during 2 h.

### Sauerbrey model application

The QCMs technique has been introduced in 1959 by Sauerbrey^41^. Basically, it relates the oscillating piezoelectric crystal frequency change to the crystal’s mass change. Recently, the QCMs have gained much interest when the quartz sensor started to be applied in liquid environments^43–45^.

Furthermore, quantifying the frequency oscillation variation of quartz in regards to the different parameters, which influence the piezoelectric crystal in liquid phase, is required in Sauerbrey model application. Under this condition, the total resonant frequency of the sensor can be expressed via the following parameters^27,46–48^:

1 $$\Delta f = \Delta f_{T} + \Delta f_{r} + \Delta f_{\eta ,\rho } + \Delta f_{m} + \Delta f_{p}$$

where ∆*f*_*T*_ stands for the temperature’s frequency variation, ∆*f*_*r*_ stands for the frequency variation corresponding to the roughness effect, ∆*f*_*η,ρ*_ is the viscosity and density variation of the solution, ∆*f*_*m*_ is the mass impact and ∆*f*_*p*_ is the pressure influence.

Taking into account the fact that:

- ∆*f*_*T*_ is neglected whenever the experiments are performed at a constant-temperature.
- ∆*f*_*r*_ is neglected whenever the used piezoelectric sensor is a polished crystal.
- ∆*f*_*η,ρ*_ is also neglected whenever the solution properties have low impacts during the experimental measurements^27,46–48^.

Therefore, whenever neglecting ∆*f*_*T*_, ∆*f*_*r*_ and ∆*f*_*η,ρ*_, the oscillation frequency of quartz turns to be:

2 $$\Delta f = \Delta f_{m} + \Delta f_{p}$$

Here, it should be underlined that the present paper focuses on the-porphyrin adsorbent metals adsorbates-interactions. So, the sensor frequency-variation should be mainly affected by the mass variation -impact ∆*f*_*m*_ without the pressure influence- intervention of ∆*f*_*p*_. This fact is considered in the following section.

### Adsorbed quantities of metals through the experimental measurements

The QCM strategy consists in presenting the sensor electrode based porphyrin. This latter is enclosed in a reactor replete with a pure water volume *V*_*s*_ of 150 mL. Through a coaxial cable, the adsorption cell is linked to the frequency-meter monitor.

It should be noted that the experimental measurements must be done after the sensor resonant frequency is stabilized. Doing so, this frequency change is discarded from the total frequency variation Δ*f*. As a result, this latter is computed considering only the mass variation’s frequency change on the adsorbing surface ∆*f*_*m*_, which yields:

$$\Delta f = \Delta f_{m}$$

Moreover, in order to instill quantities of the metals’ adsorbates solutions in the bain-marie, a micropipette is utilized. Note here that the addition were made from stock solutions of the three adsorbates (zinc chloride, nickel chloride and chromium chloride) which were prepared by dissolving the metals compounds in pure water giving solutions of concentrations varying between 10^–3^ mol L^−1^ and 0.1 mol L^−1^. Then, the matter-conservation’s law allows expressing the injected adsorbates volumes as follows:

3 $$V_{ad} = \frac{{c_{f} \times V_{s} }}{{c_{0} }}$$

After each injection, the sensor resonant frequency Δ*f*_*m*_ is measured using the frequency meter monitor. During an isotherm measurement, one can notice that the added quantities (do not exceed 2 mL) are imperceptible compared to the pure water volume *V*_*s*_ enclosed in the reactor (equal to 150 mL). The influence of the added volumes on the quartz sensor is therefore barely significant.

Depending on the relation of Sauerbrey, the complexed quantities of each metal are determined in terms of the crystal sensitivity- factor *C* (Hz cm^2^ μg^−1^) and the metal mass- variation *Δm* as follows^41,49^:

4 $$\Delta f_{m} = - C \times \Delta m$$

**Figure 2:**
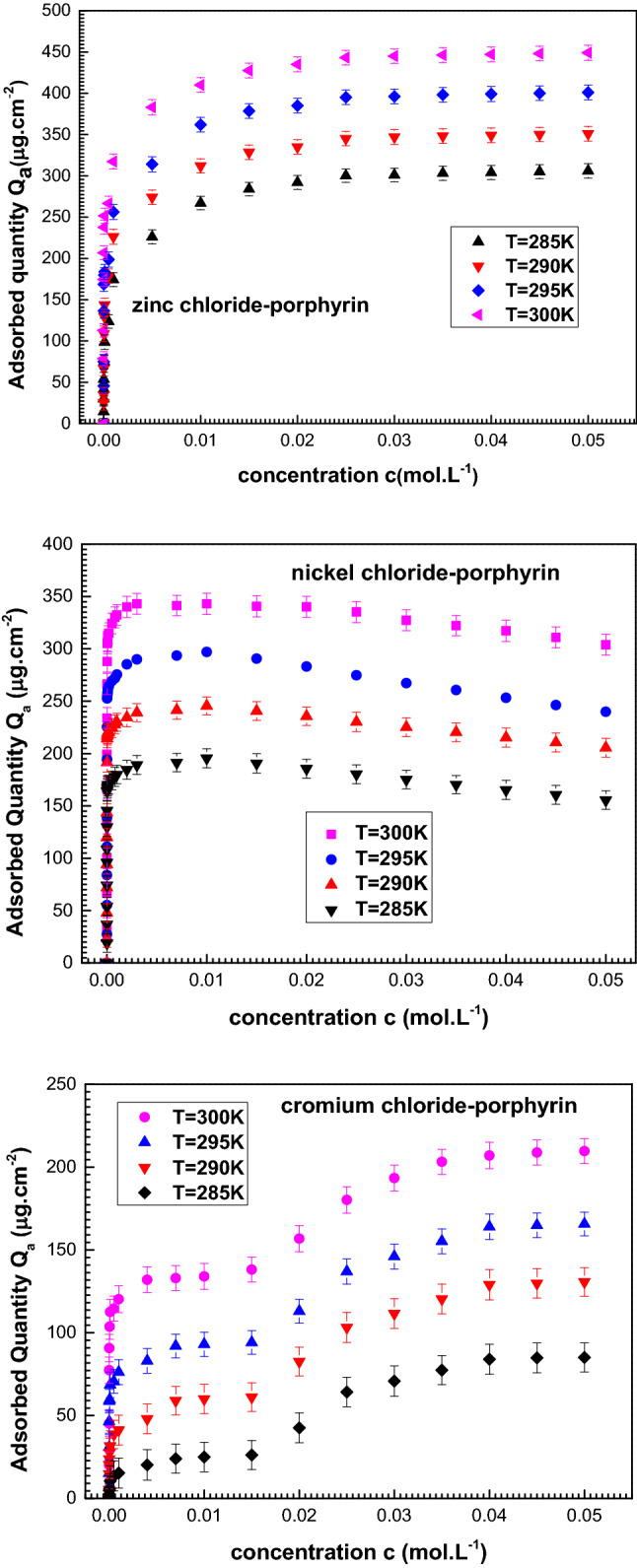
Experimental adsorption isotherms zinc chloride, nickel chloride and chromium chloride on 5,10,15,20-tetrakis(4-methylphenyl) porphyrin given at four temperatures (285–300 K).

Finally, the results depicting the metals’ adsorption on porphyrin are carried out at 285–300 K and the three adsorption systems are shown in Fig. 2.

### Experimental data’s interpretation

Through the adsorption measurements’-analysis, one can clearly notice the following observations:

The tetrakis (4-methylphenyl) porphyrin is efficient when acting as three metals’ complexing sensor in peculiar ways as regards quantity or isotherm form.

Quantitatively speaking, at T = 300 K, the maximum adsorbed quantity for each adsorbate is as follows: *Q*_*a*_ (ZnCl_2_) = 455.3 μg cm^−2^ ˃*Q*_*a*_ (NiCl_2_) = 359.8 μg m^−2^ ˃ *Q*_*a*_ (CrCl_2_) = 253.7 μg cm^−2^. One can notice that the adsorbate of zinc presents the highest adsorption values. Therefore, the zinc-porphyrin complex is the first-rate when it comes to retained quantity. Now, let us focus on the various behaviors of isotherms curves, it is noted that the metals’ nature represents the predominant complexation mechanism factor. One level of saturation is depicted for the isotherms plots of the zinc and nickel compounds. Whenever all the poprhyrinic sites are filled with the metals ions, an adsorbed metal layer is created without the participation of the anionic ions (Cl^-^). The saturation state, in the adsorption process, is attained. At high adsorbate concentration, the two mono-layer adsorptions of zinc and nickel are distinct from each other. The saturation state’s stability-of zinc is fulfilled thanks to the strong zinc-porphyrin interaction and without any inverse phenomenon even if this connection is cut. However, at high concentration the insecurity of the constructed bond between nickel and porphyrin yields the downtrend of the isotherms of the nickel compound. In fact, the formed nickel-porphyrin complex instability’s role of is due to the lateral interactions between the ions as a desorption process happened at the saturation level. It should be noted that the adsorption curves of the chromium compound revealed two states of saturation are interrelated to two formed adsorbed layers. Therefore, the chromium adsorption is done conforming to the layer by layer method^50,51^. Consequently, the development of the first layer is achieved by the adsorption of a number of cations Cr^2+^ by the porphyrins. Repulsion between the adsorbed layers will take place, if the second adsorbed layer is formed by the same charged compounds. So, resolving this electrostatic problem represents the anionic ions role. Indeed, the formulation of the second layer with the anions is dedicated to avoid the repulsion between the two layers^50,51^. For the sake of theoretical investigation of these experimental observations, let us consider now a deeper analysis.

## Advanced modeling and physicochemical interpretation

In theory, the classical equations’ use of adsorption models for the description of isotherms allows neither a deep microscopic nor a deep macroscopic interpretation of the adsorption problem. For instance, they are confined to a mono-layer process adsorption–description like the Langmuir model^32^. Erroneous depictions can be the result of such a classical model for a multi-layer process such as the chromium, which is discussed in this paper. Concerning the inspection of some parameters’ influences on adsorption like temperature, the use of classical models is not recommended^33–35^. Nevertheless, the advanced equations provide interesting interpretations. The adsorption of zinc and nickel depend upon a mono-layer model as reported by the isotherms of these systems. An LBL process is necessary for the adsorption of the chromium compound. According to the statistical physics theory, the isotherms’ modeling depends on the advanced models by selecting the most appropriate one for the isotherm’s description through its physical parameters^38,51^.

### Adsorption models’ development

Assuming that the establishment of an equilibrium between the free adsorbates phase (*A*) and the formed complexes (*A*_*n*_*-S*)-adsorbed phase is the first hypothesis of the statistical physics formalism. The equation of the equilibrium adsorption which represents the metals’ number captured by one receptor site is expressed in terms of the stochiometric variable n as follows^51–54^:

5 $$nA + \, S \rightleftharpoons A_{n} - S$$

The grand-canonical situation provides an investigation of the relation between the adsorbed phase and the adsorbates’ reservoir of the resulting complexes^52,53^. Therefore, the common partition function (*z*_*gc*_) is given by the following equation:

6 $$z_{gc} = \sum\limits_{{N_{i} }} {e^{{ - \beta \left( { - E_{i} - \mu } \right)N_{i} }} }$$

where, − *E*_*i*_ is the parameter energy and μ is the chemical potential.

Thus, an accurate selection of these parameters must be accomplished and introduced in the partition function. For the energy introduction, one energy level is to be used for a mono-layer-process and two-energies for a double-layer-mechanism. The introduction of the chemical potential of ideal gas *μ*_*p*_ or the chemical potential of real gas *μ*_*r*_ leads to mono-layer processes as well as double-layer processes.

As far as the chemical potential of ideal gas *μ*_*p*_ is concerned, the mutual interactions between the adsorbates are negligible as in the following equation^54–56^:

7 $$\mu_{p} = \frac{1}{\beta }\ln \left( {\frac{N}{{z_{Tr} }}} \right)$$

where *N* is the number of adsorbates.

However, as regards the chemical potential of real gas *μ*_*r*,_ a description of the lateral interactions is expressed in terms of the cohesion pressure *a* and the covolume *b* parameters as follows^52,54^:

8 $$\mu_{r} = \mu_{p} + \frac{1}{\beta }\ln \frac{1}{1 - bc} + \frac{1}{\beta }\frac{bc}{{1 - bc}} - 2ac$$

where *c* is the adsorbate concentration.

Considering a *P*_*M*_ identical porphyrins sites number, the average occupation number (*N*_*0*_) is determined for each model this way^54,56^:

9 $$N_{0} = \frac{{P_{M} }}{\beta }\frac{{\partial \ln (z_{gc} )}}{\partial \mu }$$

For each model, the expressions of the complexed quantity (*Q*_*a*_) corresponding to two mono layer models as well as to two double layer models, taking into account the coefficient *n* and the average occupation quantity *N*_*0*_, are given as follows^51–56^:

10 $$Q_{a} = n \times N_{0}$$

The four advanced adsorption models’ development is illustrated in Table 1.

**Table 1:** Analytical expressions of the grand-canonical partition function (*z*_*gc*_) and the adsorbed quantity (*Q*_*a*_) corresponding to the two mono-layer models of ideal gas and real gas and the two double-layers models of ideal gas and real gas.

Adsorption model	Ideal gas approach (*µ*_*p*_ (Eq. 7))	Real gas approach (*µ*_*r*_ (Eq. 8))
Mono-layer model	$z_{gc} = 1 + e^{{\beta (E + \mu_{p} )}}$ $Q_{a} = \frac{{nP_{M} }}{{1 + \left( {\frac{{c_{1/2} }}{c}} \right)^{n} }}$ where *c*_*1/2*_ is: $c_{1/2} = Se^{{ - \frac{{E_{1/2} }}{{k_{B} T}}}}$ *S*: adsorbate solubility	$z_{gc} = 1 + e^{{\beta (E + \mu_{r} )}}$ $Q_{a} = \frac{{nP_{M} }}{{1 + \left( {w_{1/2} \frac{1 - bc}{c}e^{2\beta ac} e^{{ - \frac{bc}{{1 - bc}}}} } \right)^{n} }}$ where *w*_*1/2*_ is: $w_{1/2} = Se^{{ - \frac{{E_{1/2} }}{{k_{B} T}}}}$ *S*: adsorbate solubility
Double-layer model	$z_{gc} = 1 + e^{{\beta (E_{1} + \mu_{p} )}} + e^{{\beta (E_{1} + E_{2} + 2\mu_{p} )}}$ $Q_{a} = nP_{M} \frac{{\left( {\frac{c}{{c_{1} }}} \right)^{n} + 2\left( {\frac{c}{{c_{2} }}} \right)^{2n} }}{{1 + \left( {\frac{c}{{c_{1} }}} \right)^{n} + \left( {\frac{c}{{c_{2} }}} \right)^{2n} }}$ where *c*_*1*_ and *c*_*2*_ are: $c_{1,2} = Se^{{ - \frac{{E_{1,2} }}{{k_{B} T}}}}$ _*S*: adsorbate solubility_	$z_{gc} = 1 + e^{{\beta (E_{1} + \mu_{r} )}} + e^{{\beta (E_{1} + E_{2} + 2\mu_{r} )}}$ $Q_{a} = nP_{M} \frac{{\left( {\frac{c}{{w_{1} (1 - bc)e^{2\beta ac} e^{{ - \frac{bc}{{1 - bc}}}} }}} \right)^{n} + 2\left( {\frac{c}{{w_{2} (1 - bc)e^{2\beta ac} e^{{ - \frac{bc}{{1 - bc}}}} }}} \right)^{2n} }}{{1 + \left( {\frac{c}{{w_{1} (1 - bc)e^{2\beta ac} e^{{ - \frac{bc}{{1 - bc}}}} }}} \right)^{n} + \left( {\frac{c}{{w_{2} (1 - bc)e^{2\beta ac} e^{{ - \frac{bc}{{1 - bc}}}} }}} \right)^{2n} }}$ where *w*_*1*_ and *w*_*2*_ are: $w_{1,2} = Se^{{ - \frac{{E_{1,2} }}{{k_{B} T}}}}$ _*S*: adsorbate solubility_

### Appropriate models for the isotherms description

We have been selecting via a numerical program the appropriate model based on Levenberg–Marquardt algorithm^57^. The lower the gap between the theoretical estimations and the experimental values is the better the descriptive model becomes. Therefore, in order to obtain the finest fitting result, this gap should be lower than 5%, which guarantees a confidence level of 95%^54,58^. To evaluate the four advanced models, the numerical simulation of experimental data is discussed considering three adjustment errors coefficients^51^. It is to be noted that the first error coefficient known as determination coefficient R^2^ offers the best result as its value almost equals the unit^58,59^. The second one is the AIC coefficient and the model displaying the lowest AIC-value represents the finest fitting model^51^. When its value is inferior to two, the third one, known as the non-standardized error coefficient RMSE is the best fitting model^59–62^.

The coefficients values of errors fitting are given in Tables 2, 3 and 4.

**Table 2:** Values of the correlation coefficient R^2^, the residual root mean square coefficient RMSE and the Akaike information criterion AIC deduced from fitting the experimental adsorption isotherms of ZnCl_2_ on 5,10,15,20-tetrakis(4-methylphenyl) porphyrin with the four advanced models.

Adsorption system	Zinc chloride-porphyrin			
Temperature (K)	285	290	295	300
**Mono-layer model (ideal gas)**				
R^2^	0.99 (± 0.006)	0.99 (± 0.008)	0.98 (± 0.009)	0.97 (± 0.007)
RMSE	1.51 (± 0.4)	1.66 (± 0.2)	1.02 (± 0.4)	1.4 (± 0.1)
AICAIC	15.88 (± 1.8)	16.4 (± 0.8)	17.4 (± 1.1)	16.26 (± 2.4)
**Mono-layer model (real gas)**				
R^2^	0.89 (± 0.03)	0.87 (± 0.02)	0.88 (± 0.01)	0.89 (± 0.01)
RMSE	3.01 (± 0.8)	3.99 (± 0.3)	3.5 (± 0.7)	3.64 (± 0.87)
AIC	20.7 (± 2. 3)	21.5 (± 2. 5)	21.5 (± 1. 69)	20.99 (± 2. 01)
**Double-layer model (ideal gas)**				
R^2^	0.81 (± 0.02)	0.84 (± 0.05)	0.83 (± 0.03)	0.79 (± 0.04)
RMSE	5.2 (± 0.89)	5.33 (± 0.05)	6.7 (± 0.1)	6.2 (± 0.3)
AIC	24.6 (± 1.5)	23.98 (± 1.8)	24.79 (± 2.05)	25.4 (± 2.4)
**Double-layer model (real gas)**				
R^2^	0.81 (± 0.05)	0.81 (± 0.03)	0.76 (± 0.09)	0.77 (± 0.03)
RMSE	7.1 (± 1.2)	7.9 (± 2.08)	6.98 (± 1.79)	7.21 (± 2.4)
AIC	26.7 (± 1.54)	27.46 (± 2.49)	28.9 (± 3.7)	27.84 (± 3.2)

**Table 3:** Values of the correlation coefficient R^2^, the residual root mean square coefficient RMSE and the Akaike information criterion AIC deduced from the numerical adjustment of experimental isotherms of NiCl_2_ on 5,10,15,20-tetrakis(4-methylphenyl) porphyrin with the four statistical physics models.

Adsorption system	Nickel chloride-porphyrin			
Temperature (K)	285	290	295	300
**Mono-layer model (ideal gas)**				
R^2^	0.81 (± 0.03)	0.77 (± 0.09)	0.82 (± 0.02)	0.83 (± 0.01)
RMSE	6.66 (± 0.69)	7.3 (± 0.92)	6.91 (± 0.81)	7.45 (± 0.89)
AIC	29.1 (± 3.7)	31.6 (± 4.16)	30.67 (± 4.2)	29.42 (± 3.67)
**Mono-layer model (real gas)**				
R^2^	0.99 (± 0.005)	0.97 (± 0.009)	0.98 (± 0.007)	0.98 (± 0.004)
RMSE	1.66 (± 0.1)	1.78 (± 0.09)	1.22 (± 0.5)	1.45 (± 0.2)
AIC	19.4 (± 1.52)	20.7 (± 1.51)	19.47 (± 1.34)	19.67 (± 1.74)
**Double-layer model (ideal gas)**				
R^2^	0.81 (± 0.02)	0.82 (± 0.03)	0.78 (± 0.05)	0.82 (± 0.01)
RMSE	4.8 (± 0.55)	3.29 (± 0.12)	5.21 (± 0.43)	4.95 (± 0.5)
AIC	26.6 (± 1.99)	26.8 (± 1.39)	27.91 (± 2.7)	26.3 (± 1.64)
**Double-layer model (real gas)**				
R^2^	0.84 (± 0.04)	0.85 (± 0.02)	0.79 (± 0.04)	0.8 (± 0.05)
RMSE	5.9 (± 1.64)	4.89 (± 0.36)	6.02 (± 1.9)	5.82 (± 1.88)
AIC	28.7 (± 2.7)	29.31 (± 3.26)	28.5 (± 3.5)	27.28 (± 3.07)

**Table 4:** Values of the correlation coefficient R^2^, the residual root mean square coefficient RMSE and the Akaike information criterion AIC deduced from the numerical adjustment of experimental isotherms of CrCl_2_ on 5,10,15,20-tetrakis(4-methylphenyl) porphyrin with the statistical physics models.

Adsorption system	Chromium chloride-porphyrin			
Temperature (K)	285	290	295	300
**Mono-layer model (ideal gas)**				
R^2^	0.71 (± 0.039)	0.77 (± 0.09)	0.62 (± 0.05)	0.73 (± 0.041)
RMSE	8.67 (± 2.67)	8.12 (± 2.02)	7.98 (± 1.89)	7.56 (± 1.16)
AIC	32.7 (± 1.51)	34.8 (± 2.52)	34.78 (± 1.92)	35.76 (± 2.54)
**Mono-layer model (real gas)**				
R^2^	0.79 (± 0.063)	0.79 (± 0.06)	0.78 (± 0.07)	0.78 (± 0.059)
RMSE	5.47 (± 1.19)	6.9 (± 2.05)	7.34 (± 2.29)	6.57 (± 1.51)
AIC	32.4 (± 2.55)	34.7 (± 3.07)	35.59 (± 1.85)	33.12 (± 2.41)
**Double-layer model (ideal gas)**				
R^2^	0.98 (± 0.006)	0.99 (± 0.002)	0.98 (± 0.004)	0.97 (± 0.006)
RMSE	2.08 (± 0.014)	1.85 (± 0.065)	1.91 (± 0.048)	1.95 (± 0.049)
AIC	26.94 (± 1.78)	26.82 (± 1.39)	26.47 (± 3.04)	27.41 (± 2.45)
**Double-layer model (real gas)**				
R^2^	0.89 (± 0.015)	0.91 (± 0.007)	0.90 (± 0.005)	0.89 (± 0.009)
RMSE	5.79 (± 0.25)	6.78 (± 1.34)	5.94 (± 1.19)	5.93 (± 0.94)
AIC	30.67 (± 0.08)	31.92 (± 0.19)	30.75 (± 0.06)	31.98 (± 1.14)

According to Table 2, the description of the isotherms of zinc requires the ideal gas mono-layer model use as it displays the finest fitting criterion. As far as the isotherms of nickel are concerned, the mono layer model of real gas gives the best coefficients values of adjustment (Table 3). This way, the lateral interactions between the adsorbates represent the reason of the isotherms’ drop at saturation. The double layer model of ideal gas represents the best fitting model for the description of the isotherms of chromium according to the selection criteria (Table 4). So, a layer by layer process happens between the cations (Cr^2+^) and the anions (Cl^−^).

**Figure 3:**
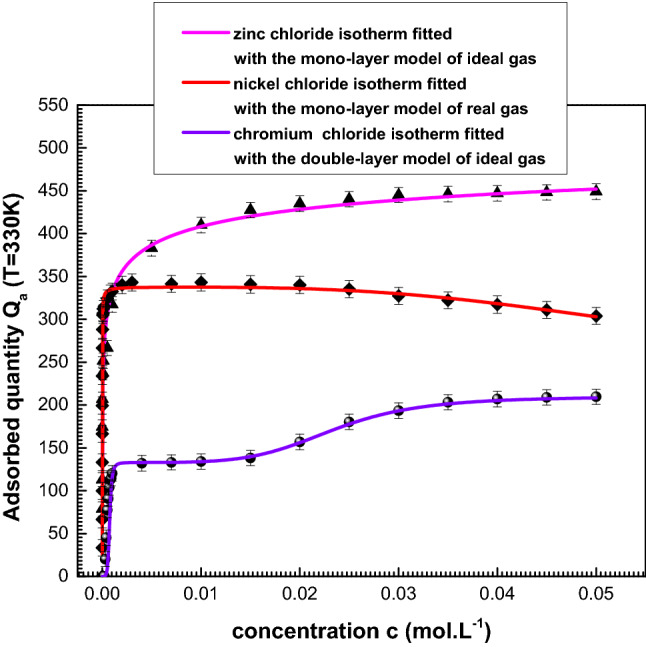
Experimental adsorption isotherms simulated with the adequate statistical physics model at 300 K.

At T = 300 K, the experimental isotherms along with the theoretical simulations are compared to the appropriate model in Fig. 3: the ideal gas mono-layer model matches with the experimental data of zinc. The ideal gas mono-layer model promotes the matching of nickel-adsorption isotherms. Whereas, the ideal gas double layer model is matched to the chromium experimental data.

In the next section, the three complexation mechanisms’ microscopic investigation is expanded referring to the adopted models’ fitting parameters-values.

### Models’ parameters and physicochemical discussion

The chosen models for the isotherms-curves’ description, as seen in Table 1, include steric parameters and energetic variables. By steric parameters, we mean the metals-number per porphyrin site *n* and the occupied adsorbent sites number *P*_*M*_. By energetic variables, we mean *c*_*1/2*_ for zinc, *w*_*1/2*_ for nickel and *c*_*1*_ and *c*_*2*_ for chromium. The description of nickel adsorption is done by the cohesion pressure *a* and the covolume *b* which represent the lateral interactions variables.

The adsorption systems’ parameters values are given in Table 5.

**Table 5:** Fitting values of the physicochemical parameters (*n, P*_*M*_*, a, b, c*_*1/2*_*, w*_*1/2*_*, c*_*1*_ and *c*_*2*_) deduced from the adjustment of experimental isotherms of ZnCl_2_, NiCl_2_ and CrCl_2_ with the three adopted models.

Adsorption isotherm/fitting model	Models’ parameters	T = 285 K	T = 290 K	T = 295 K	T = 300 K
ZnCl_2_/mono-layer model (Ideal gas)	*n*	0.75	0.84	0.91	0.94
	*P*_*M*_	299.2	351.6	379.5	421.4
	*c*_*1/2*_	0.007	0.008	0.009	0.008
NiCl_2_/mono-layer model (Real gas)	*n*	0.55	0.64	0.72	0.84
	*P*_*M*_	239.6	259.2	317.5	355.2
	*w*_*1/2*_	0.006	0.0067	0.0073	0.0069
	*a* (× 10^–9^)	9.9	8.2	7.6	7.13
	*b* (× 10^–12^)	1.4	2.1	3.8	4.4
CrCl_2_/double-layer model (Ideal gas)	*n*	0.41	0.52	0.64	0.73
	*P*_*M*_	147.9	184.8	239.5	279.4
	*c*_*1*_	0.0029	0.0032	0.0034	0.0031
	*c*_*2*_	0.026	0.025	0.024	0.025

#### Steric parameters n and P_M_ and lateral interactions’ parameters a and b

The steric parameters pave the way for a comparison between the performances of the three systems as far as quantity is concerned^63^. According to Table 5 and at a temperature T equal to 300 K, it is noticeable that:

$$n({\text{ZnCl}}_{{2}} ) = 0.{94} > n({\text{NiCl}}_{{2}} ) = 0.{84} > n({\text{CrCl}}_{{2}} ) = 0.{73}$$

$$P_{M} ({\text{ZnCl}}_{{2}} ) = {421}.{4 }\mu {\text{g}}/{\text{cm}}^{{2}} > P_{M} ({\text{NiCl}}_{{2}} ) = {355}.{2 }\mu {\text{g}}/{\text{cm}}^{{2}} > P_{M} ({\text{CrCl}}_{{2}} ) = {279}.{4 }\mu {\text{g}}/{\text{cm}}^{{2}}$$

A fitted values comparison between the metals-number per porphyrin site *n* and the occupied sites *P*_*M*_ affirms that the porphyrinic surface, which is provided by the complexed quantity of zinc, is better than other metals as far as quantity is concerned.

One can say that all the adsorption systems have values of *n* inferior to the unit, which proves that the multi-docking mechanism is the origin of the three ions complexation (the charged metal ions cannot be aggregated in one receptor site of porphyrin because of the repulsion interaction)^53^. When we follow the order in which the values of *P*_*M*_ occur, we can notice that both the zinc and the nickel were added promptly because only one layer is constructed. In the meanwhile, the anionic ions did not contribute to this formation. In fact, in the case of the anionic ions’ participation (chromium chloride adsorption), the porphyrins’ complexation would have been disfavored by reason of the interaction between the two adsorbed layers.

A lateral interactions’ description, by means of *a* and *b*, is presented by the real gas model which aims at inspecting the adsorption of the nickel compound^64^. If we compare the nickel-compound complexation to the zinc process, we notice that it is disfavored by the lateral adsorbate–adsorbate interaction depicted by the variables mentioned above.

#### Energetic parameters

Each system’s adsorption energies can be calculated by the energy-expression of each adopted model including the energetic parameters’ fitted values: *c*_*1/2*_ for zinc, *w*_*1/2*_ for nickel and *c*_*1*_ and *c*_*2*_ for chromium, (see Table 1)^54,64^.

Table 6 expresses the three systems’ calculated energies.

**Table 6:** Values of the adsorption energies |− *E*_*1/2*_| for zinc and nickel adsorptions and |− *E*_*1*_| and |− *E*_*2*_| for chromium adsorption given in modulus values at 285, 290, 295 and 300 K.

Adsorption system	Adsorption energy	285 K	290 K	295 K	300 K
ZnCl_2_-porphyrin	|− *E*_*1/2*_| (kJ mol^−1^)	53.1	62.4	65.1	69.5
NiCl_2_-porphyrin	|− *E*_*1/2*_| (kJ mol^−1^)	32.9	36.8	38.1	40.8
CrCl_2_-porphyrin	|− *E*_*1*_| (kJ mol^−1^)	25.2	28.4	33.4	34.5
	|− *E*_*2*_| (kJ mol^−1^)	16.9	18.8	20.3	20.9

According to Table 6, it is noticeable that the energies’ order in modulus values is as follows:

$$| - E_{1/2} | \, ({\text{ZnCl}}_{{2}} ) = {69}.{\text{5 kJ }}/{\text{mol }} > | - E_{1/2} | \, ({\text{NiCl}}_{{2}} ) = {4}0.{\text{8 kJ }}/{\text{mol }} > \left| { - E_{1} } \right| \, ({\text{CrCl}}_{{2}} ) \, = { 34}.{\text{5 kJ }}/{\text{mol}} > \, \left| { - E_{2} } \right| \, ({\text{CrCl}}_{{2}} ) = {2}0.{\text{9 kJ }}/{\text{mol}}$$

The zinc-adsorption possesses the maximal energies-values, then, the zinc-porphrin complex is displayed as the best complex as far as stability is concerned when compared to the complexes of nickel and chromium. Also, the adsorption energy values, shown in the adsorption process of zinc, are beyond 40 kJ mol^−1^, which implies that the chemisorption process happened throughout the zinc-complexation^53,65,66^. Zinc-porphyrin bond can be either covalent or ionic^66^. In order to establish this kind of bonds, a change in terms of particles’ structures as well as an electron density- rearrangement between the adsorbent and the adsorbate should take place. So, a multi-layer process can neither result from the chemical adsorption nor be influenced by the lateral interactions^65,66^. The nickel and chromium processes, on the other hand, display adsorption energies results below 40 kJ mol^−1^, which implies that a physical adsorption process between the two metals and the adsorbent took place^5,65^. The van der Waals forces along with hydrogen bonding, which are physical bonds, can be created between both metals and the porphyrin cavities^5,65,66^. Because there is no rearranged electron density of the adsorbent and the adsorbate, a linkage like this is slightly energetic. Because of the feeble adsorbate adsorbent complex binding, a desorption mechanism could happen as the nickel-adsorption case and a multi-layer-mechanism could be produced as in the chromium case.

### Theoretical results’ discussion

**Figure 4:**
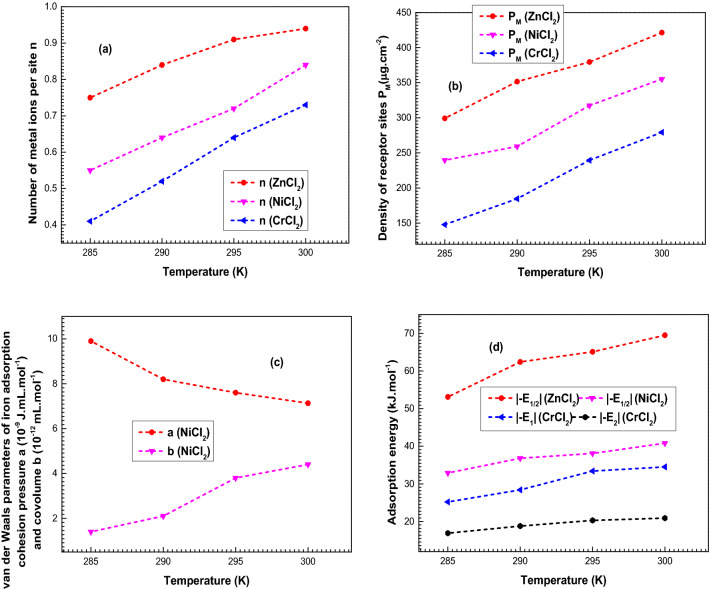
Evolutions of the two steric parameters (**(a)** the number of metal ions per site **n** and **(b)** the density of receptor porphyrins sites **P**_**M**_), the lateral interactions parameters **(c)** (cohesion pressure **a** and covolume **b**) and the adsorption energies **(d)** (|− E_1/2_|, |− E_1_| and |(− E_2_)|) as a function of temperature (285–300 K).

The diversity of all the physicochemical variables versus temperature is shown in Fig. 4. Figure 4a illustrates the evolution of the number of metals caught by one porphyrin. Figure 4b indicates the variation of the receptor sites density *P*_*M*_. Figure 4c presents the evolution of the lateral interactions parameters *a* and *b*. Figure 4d reveals the variation of the adsorption energies.

Referring to these figures, it is to be noted that the steric parameters’ fitted values *n* and *P*_*M*_ as well as the adsorption energies values’ increase is associated with a temperature’s increase. Thus, the adsorption is an endothermic process for all the systems.

Broadly, adsorption is an exothermic process where the adsorption reaction is hindered by the increase of temperature. This implies that at one pressure or concentration, the adsorbed amount decreases as the temperature increases. Nevertheless, it can be seen from the adsorption isotherms, illustrated in Fig. 2, that the adsorbed amounts increase with an increase of the temperature from 285 to 300 K. Figure 4b explains that the density of receptor sites *P*_*M*_ heightened hand in hand with temperature for the three systems. Along with the increase of temperature, new receptor pophyrins sites hidden beforehand when the temperature was low, appear and take part in the adsorption process, increasing thus both the adsorbed quantity and the adsorption energies (Fig. 4d)^36,37^. For the nickel adsorption, it is noticeable that the increase of *b* is accompanied by an increase in temperature along with an increase in the adsorption energies and the steric variables. Whereas, when the temperature rises, the cohesion pressure decreases (Fig. 4c), which reveals that the lateral adsorbate adsorbate interactions are disfavored by the rise of temperature as it widens the distance between the adsorbates^38,64^. Moreover, the cohesion pressure utility is only considered in order to interpret the decrease in adsorbed amount at saturation. While, the others variables have proved their decisive roles in the adsorption procedure by showing temperature behaviors close to the complexed quantity.

## Thermodynamics

A thermodynamic study makes the macroscopic interpretation possible. Both entropy and free enthalpy expressions can be calculated according to these formulas^52,55^:

11 $$S{}_{a} = - \frac{{P_{M} }}{T}\frac{{\partial \ln z_{gc} }}{\partial \beta } + k_{B} P_{M} \ln z_{gc}$$

12 $$G = \mu \times Q_{a}$$

The thermodynamic functions are expressed in Table 7.

**Table 7:** Analytical expressions of the entropy (*S*_*a*_*/k*_*B*_) and the free enthalpy (*G/k*_*B*_*T*) of the two mono-layer adsorptions of zinc and nickel and the L.B.L double-layer adsorption of chromium.

Adsorption system	Entropy *S*_*a*_*/k*_*B*_	Free Enthalpy *G/k*_*B*_*T*
ZnCl_2_	$P_{M} \left( {\ln (1 + (\frac{c}{{c_{1/2} }})^{n} ) - \frac{{(\frac{c}{{c_{1/2} }})^{n} \ln (\frac{c}{{c_{1/2} }})^{n} }}{{1 + (\frac{c}{{c_{1/2} }})^{n} }}} \right)$	$nP_{M} \left( {\ln (\frac{c}{{z_{tr} }})} \right).\left( {\frac{{(\frac{c}{{c_{1/2} }})^{n} }}{{1 + (\frac{c}{{c_{1/2} }})^{n} }}} \right)$
NiCl_2_	$$\begin{gathered} P_{M} \ln \left( {1 + \left( {\frac{c}{{w_{1/2} (1 - bc)e^{2\beta ac} e^{{ - \frac{bc}{{1 - bc}}}} }}} \right)^{n} } \right) - \hfill \\ P_{M} \frac{{\left( {\frac{c}{{w_{1/2} (1 - bc)e^{2\beta ac} e^{{ - \frac{bc}{{1 - bc}}}} }}} \right)^{n} \ln \left( {\frac{c}{{w_{1/2} (1 - bc)e^{2\beta ac} e^{{ - \frac{bc}{{1 - bc}}}} }}} \right)^{n} }}{{1 + \left( {\frac{c}{{w_{1/2} (1 - bc)e^{2\beta ac} e^{{ - \frac{bc}{{1 - bc}}}} }}} \right)^{n} }} \hfill \\ \end{gathered}$$	$$\begin{gathered} nP_{M} \left( {\ln \left( {\frac{c}{{z_{tr} }}} \right) + \ln \frac{1}{1 - bc} + \frac{bc}{{1 - bc}} - \frac{2ac}{{k_{B} T}}} \right). \hfill \\ \left( {\frac{{\left( {\frac{c}{{w_{1/2} (1 - bc)e^{2\beta ac} e^{{ - \frac{bc}{{1 - bc}}}} }}} \right)^{n} }}{{1 + \left( {\frac{c}{{w_{1/2} (1 - bc)e^{2\beta ac} e^{{ - \frac{bc}{{1 - bc}}}} }}} \right)^{n} }}} \right) \hfill \\ \end{gathered}$$
CrCl_2_	$P_{M} \left( {\ln \left( {1 + \left( {\frac{c}{{c_{1} }}} \right)^{n} + \left( {\frac{c}{{c_{2} }}} \right)^{n} } \right) - \frac{{\left( {\frac{c}{{c_{1} }}} \right)^{n} \ln \left( {\frac{c}{{c_{1} }}} \right)^{n} + \left( {\frac{c}{{c_{2} }}} \right)^{n} \ln \left( {\frac{c}{{c_{2} }}} \right)^{n} }}{{1 + \left( {\frac{c}{{c_{1} }}} \right)^{n} + \left( {\frac{c}{{c_{2} }}} \right)^{n} }}} \right)$	$$\begin{gathered} nP_{M} \left( {\ln (\frac{c}{{z_{tr} }})} \right) \hfill \\ \left( {\frac{{(\frac{c}{{c_{1} }})^{n} + 2(\frac{c}{{c_{2} }})^{2n} }}{{1 + (\frac{c}{{c_{1} }})^{n} + (\frac{c}{{c_{2} }})^{2n} }}} \right) \hfill \\ \end{gathered}$$

**Figure 5:**
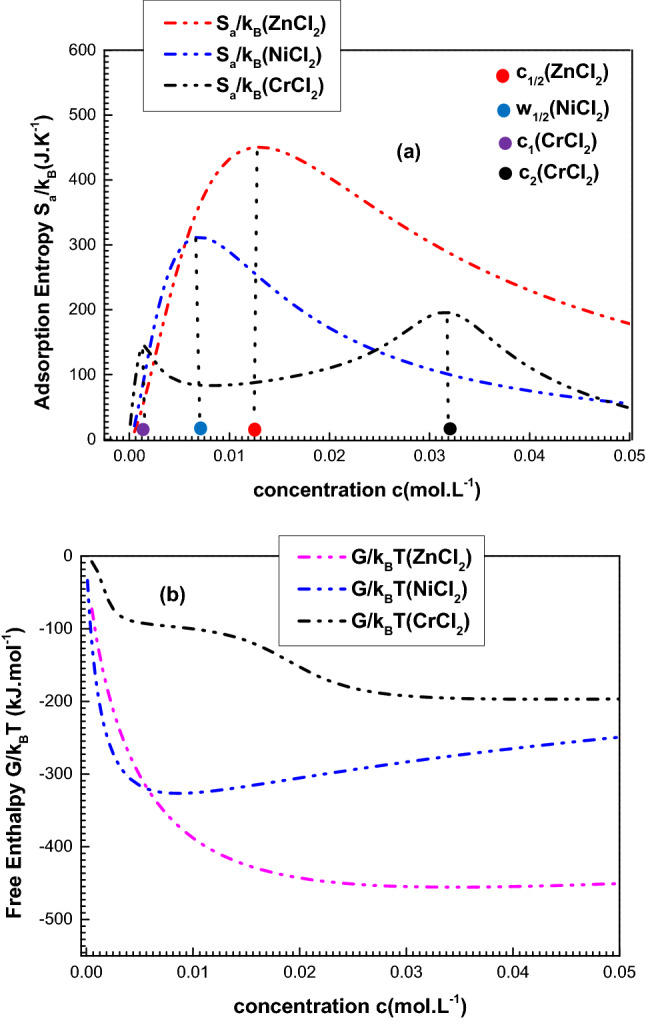
Evolutions of the entropy **(a)** and the free enthalpy **(b)** versus concentration for the three adsorption systems at 300 K.

Figure 5 shows the two thermodynamic functions versus the equilibrium concentration for the three adsorption systems.

Figure 5a, illustrating the evolution of the entropy, reveals that this latter attains its maximal value as far as the adsorbate concentration is equal to the energetic parameter-value. Thus, the highest disorder is reached at these specific points^52^. In addition, it is worth saying that the entropy of the zinc and nickel systems reveals one peak as one level of energy allows the adsorption of these compounds on the porphyrins. Whereas, two levels of energy permit an interpretation of chromium adsorption, which explains the two maximums. It is noticeable that peak number 1, which coincides with the partial disorder of the first layer adsorption, is less than peak number 2, which echoes the total disorder of the whole system^63^.

Figure 5b displays the variation of the free enthalpy. It shows that the enthalpy values are negative, which proves that the three metals’ adsorptions have automatically progressed so as to reach the saturation state. In addition, the enthalpy of the zinc compound is in steady state at high concentration. However, the algebraic value of the nickel compound is amplified at saturation, which means that the adsorption process of NiCl_2_ was hindered by the lateral interactions at high concentration^54^. On the other hand, CrCl_2_ presents two stability states. A first one consists of the first layer saturation and a second one coincides with the two formed layers’ total saturation^63^.

## Conclusion

In this paper, the spin coating technique has been demonstrated to be a utile procedure for the performance of quartz disc sensor with macrocyclic systems. This research has surveyed the piezoelectric sensor based porphyrin film as a complexing adsorbent of three metals paving the way for the experimental adsorption isotherms quantification. Through the isotherm plots analysis, it has been found that the zinc-porphyrin complex is the most fitted on the subject of quantity as well as stability. The lateral interactions of adsorbates have altered the firmness of the formed nickel-porphyrin complex, which results in a reversible mechanism at saturation. The layer by layer procedure of chromium adsorption has revealed the role of anionic ions (that is chloride). The complexation of chromium on the porphyrinic surface is disfavored by the contribution of this anion in the adsorption process so as the established complex stability is influenced. The isotherms’ modeling, via physical models, show that the porphyrins’ complexation stands for a multi docking mechanism and the zinc-porphyrin is the best created complex as far as amounts as well as steadiness are concerned. The adsorption of the zinc compound was proven to be a chemical process thanks to the calculation of the three systems energies. Similarly, it was demonstrated that the complexation of the nickel and chromium occurred thanks to a physical mechanism. The existence of a nickel desorption process and the creation of two layers for chromium are due to the some physical mechanisms. The analysis of the impact of temperature on the adsorption has indicated that the participation of new porphyrins sites in the adsorption is favored by the temperature rise, which reveals the endothermic nature of the three metals adsorption. Due to the exploration of the adsorption entropy, the thermodynamic evaluation has pointed out that for the three mechanisms the summit of the disorder is attained at the energetic parameters levels. It was proven that the behavior of the enthalpy determined the three complexation mechanisms’ spontaneity. One stability state, coinciding with the saturation of the adsorbed layer of the mono-layer process was provided by the nickel and zinc adsorption processes. On the other hand, two stability states were established by the double-layer adsorption process of the chromium (by partial stability level, we mean the first layer saturation and the total stability state for the full system).

### Analogy with the latest papers

The isotherms’ modeling via the advanced statistical physics yielded to impressive explanations of the adsorption mechanism regarding the established layers number (mono-layer/double-layers), the steric aspect (multi-docking/multi-ionic), the adsorption nature (exothermic/endothermic) and the created complex type (chemical/physical). The earlier conventional models like Langmuir^32^, Freundlich^33^ and others^34,35^, could not deduct these microscopic conclusions. As far as the adsorption of metals on porphyrins is concerned, precedent works considering the adsorption of FeCl_2_, MnCl_2_ and MgCl_2_ on porphyrins^51,54,64^ have demonstrated that the presence of chloride ions in the adsorbate compounds did not have any influence over the complexation process, which leads to a mono-layer adsorption phenomenon for all the metals. Others works pointed out that the layer by layer process or multi-layer ionic adsorption can only occur in the case of the metals compounds comprising the nitrate ions in their structures such as Co(NO_3_)_2_ and Mn(NO_3_)_2_^38,54^. In this work, it has been proven that the anionic ions did not participate in the zinc and nickel adsorption. Whereas, with the chromium, the chloride ions did contribute to the layer by layer adsorption process of porphyrin.

## Outlooks

Some characteristics concerning the adsorption cell’s surface morphology such as SFM and SEM methods should provide us with ameliorated results in the future papers. The density functional theory also known as DFT method where the electron localization function (ELF) and electron densities plots are presented and inspected exhaustively, can be of much use for the investigation of the complexation process of porphyrin. In addition, an amelioration in the statistical physics modeling is possible if we consider the adsorbate dissolution at the adsorption time as the current investigation looks at the adsorbate as being entirely neutral. Up to now, the ionic nature of the adsorbate in the solution has not been taken into consideration. It is crucial to accomplish this investigation, as it will be conceived as an electrochemical task. The statistical physics treatment performed by our research team did not take into consideration this fact. Our research is decisive as it scrutinizes the ionic natures of the anions as well as of the cations in the adsorption procedure. It is worth mentioning that this process is to be regarded as a binary adsorption performed by statistical physics formalism^53^. The formed charge zone is to be seen as a main influence on the adsorption process, which represents one of the outlooks of our teamwork. Moreover, the study of the aggregation of porphyrins can be one of the most interesting perspectives which should be deeply investigated because the change in the microscopic characteristics of porphyrins led to their application in numerous fields of research. Thus, the effect of aggregation for different metal ions will be discussed in future papers.

## Data Availability

The data that supports the findings of this study are available within the article.
